# Effectiveness of an online food shopping intervention to reduce salt purchases among individuals with hypertension – findings of the SaltSwitch Online Grocery Shopping (OGS) randomised trial

**DOI:** 10.1186/s12966-024-01700-9

**Published:** 2024-12-30

**Authors:** Jason HY Wu, Damian Maganja, Liping Huang, Kathy Trieu, Fraser Taylor, Eden M. Barrett, Clare Arnott, Xiaoqi Feng, Aletta E. Schutte, Gian Luca Di Tanna, Cliona Ni Mhurchu, Adrian J. Cameron, Mark D. Huffman, Bruce Neal

**Affiliations:** 1 The George Institute for Global Health, University of New South Wales, Level 18, International Towers 3, 300 Barangaroo Ave, Sydney, NSW 2000 Australia; 2 School of Population Health, University of New South Wales, Samuels Building, Samuel Terry Ave, Kensington, NSW 2052 Australia; 3 Department of Cardiology, Royal Prince Alfred Hospital, 50 Missenden Rd, Camperdown, NSW 2050 Australia; 4 Department of Business Economics, Health & Social Care, University of Applied Sciences and Arts of Southern Switzerland, Stabile Piazzetta, Via Violino 11, Manno, 6928 Switzerland; 5 Department of Epidemiology and Biostatistics, School of Population Health, University of Auckland, Building 507, 22-30 Park Avenue, Grafton, 1023 New Zealand; 6 Global Centre for Preventive Health and Nutrition, Institute for Health Transformation, Deakin University, 1 Gheringhap Street, Geelong, VIC 3220 Australia; 7 Department of Medicine, Washington University in St Louis, 660 S. Euclid Ave, St. Louis, MO 63110-1010 USA; 8 Department of Epidemiology and Biostatistics, Imperial College London, Medical School Building, St Mary’s Campus, Norfolk Place, London, W2 1PG UK; 9 PO Box M201, Missenden Rd, Sydney, NSW W2 1PG Australia

**Keywords:** Hypertension, Sodium, Online shopping, Product labelling, Nudge

## Abstract

**Background:**

Online grocery shopping is a growing source of food purchases in many countries. We investigated the effect of nudging consumers towards purchases of lower sodium products using a web browser extension.

**Methods:**

This trial was conducted among individuals with hypertension who shopped for their groceries online in Australia. From July 2021 to June 2023, participants were randomised to use the SaltSwitch Online Grocery Shopping web browser extension or continue their usual grocery shopping for 12 weeks. The SaltSwitch extension modified a retailer’s online shopping interface to suggest similar but lower sodium alternative products to those initially selected. The primary outcome was the difference in mean sodium density (mg sodium per 1000 kcal of energy) of packaged food purchases between the intervention and control groups.

**Results:**

We randomised 185 participants of average age 56.0 (SD 11.0) years. Most were women (64%), White (89%), had BMI > 25 kg/m^2^ (91%), and were taking anti-hypertensive medication (83%). Demographic and medical characteristics were similar across the randomised groups. 182 (98%) completed the trial. Over the 12-week intervention, the sodium density of groceries purchased by the intervention group compared to the control group was 204 mg/1000 kcal lower (95%CI, -352 to -56) (*P* = 0.01). The reduction in sodium density of purchases was apparent in weeks 1–4 and sustained through the end of the trial. 86% of participants in the intervention group made at least one switch to a lower sodium product. There were no detectable effects on blood pressure, spot urine sodium concentration, or other secondary outcomes across the 12-week study period.

**Conclusions:**

Online shopping platforms provide a novel opportunity to support purchases of lower sodium foods. While the reductions in sodium density of purchases were moderate in size, population health benefits could nonetheless be large if they were sustained over time and at scale, with large and growing numbers of online grocery shoppers and a high prevalence of elevated blood pressure amongst adults.

**Trial registration:**

ACTRN12621000642886.

**Supplementary Information:**

The online version contains supplementary material available at 10.1186/s12966-024-01700-9.

## Background

High blood pressure contributes to more than 10 million premature deaths each year [1] and hundreds of billions of dollars in associated healthcare costs and lost productivity [2–4]. Excess dietary sodium is one of the primary modifiable dietary causes of hypertension [5] and reducing sodium intake is recommended by the World Health Organization (WHO) as a critical and cost-effective public health intervention that should be prioritised [6, 7]. However, dietary sodium intake remains high both among those with or without hypertension, with most adults around the world consuming twice the recommended maximum [8, 9].

Dietary sodium generally comes from two major sources – manufactured and packaged food products containing salt (sodium chloride), or salt added during cooking or at the table (so-called discretionary salt). In most high-income countries but also increasingly for low- and middle-income countries, packaged foods contribute the majority of sodium intake [10]. Given the wide variability in sodium levels that have been observed among otherwise comparable packaged food products [11–14], a promising approach for sodium reduction is to ‘switch’ to similar foods containing less sodium. However, prior research indicates that consumers struggle to locate, interpret, and use detailed, quantified nutrition labels [15, 16].

Accumulating evidence suggests that nutrition labels that interpret detailed nutrition data to provide easy-to-understand information (for instance, via colour-coding or summary indicators) [17–19] could ‘nudge’ consumers towards selecting and purchasing healthier foods [20–23], especially if shown at the point-of-purchase [24]. Further, people with diet related chronic diseases may be particularly motivated to make such changes [25–29].

Online grocery shopping is becoming increasingly popular and this setting has potential to improve consumer dietary patterns for large numbers. Online grocery purchases already account for nearly one-quarter of food retail sales in some countries [30] with the shift from in-store to online having further accelerated since the COVID-19 pandemic [30]. The marketing strategies used in online grocery food environments may predispose consumers towards less healthy products, for instance through promotions using placement- or price-based techniques, inconsistent provision of nutrition information, or the use of behavioural nudges such as arbitrary time-limited offers [31–34]. The setting also offers opportunities to improve consumer choices that are not available in physical store settings – for instance, ‘product swap’ functions targeting healthier options can be more readily implemented, and in real time, online. Few other interventions involving product swaps and/or interpretive labels in real-world online supermarkets have been reported to date, however [35–37].

Here, we describe the findings of a randomised controlled trial that investigated the effectiveness of an innovative online platform designed to nudge consumers towards lower sodium products via interpretive labelling and product swap functions, called SaltSwitch for Online Grocery Shopping (SaltSwitch OGS) [38].

## Methods

### Study design

The SaltSwitch OGS study was a two-arm, parallel-group, individual randomised, controlled trial (Additional File 1, Supplementary Fig. 1); a trial protocol has previously been published [38]. The trial received ethics approval from the University of New South Wales Human Research Ethics Committee (HC200970) and was registered on the Australian and New Zealand Clinical Trials Registry prior to study commencement (ACTRN12621000642886).

When trial recruitment began in July 2021, the primary objective was to measure the difference in mean systolic blood pressure between randomised groups, with a planned sample size of 1,966 participants. Due to significant difficulties in recruitment, in August 2022 we revised the primary objective to the difference in mean sodium density (mg sodium per 1000 kcal of energy) of packaged groceries purchased between randomised groups. Systolic blood pressure was retained as a secondary outcome. All changes were finalised before completion of recruitment and unblinding of data and documented a priori in a statistical analysis plan posted on the Open Science Framework (https://osf.io/347ps/).

### Hypothesis

We tested a primary null hypothesis of no difference in the mean sodium density of packaged groceries purchased between the intervention and control groups over 12 weeks.

### Recruitment, consent and baseline data collection

Participants were enrolled from across Australia using trial recruitment services provided by Trialfacts (www.trialfacts.com), HealthMatch (https://healthmatch.io/) and Join Us (www.joinus.org.au), as well as through advertising on social media and Google. All trial processes, commencing with provision of the Participant Information Statement and completion of the Consent Form, were done online through a bespoke study website. There was an opportunity for potential participants to speak with a study staff member during the consent process to obtain additional explanation if required. Once informed consent was obtained, a screening survey was done to assess eligibility.

### Participant inclusion and exclusion criteria

The criteria for participants to enter the trial run-in phase were: (1) adult aged 18 to 75 years living in Australia; (2) self-reported diagnosis of hypertension; (3) no dose change for the past 6 months if using anti-hypertensive medication; (4) self-reported intent to shop online for most groceries using a computer for the duration of the study; and (5) provide informed consent. People were excluded if another household member was already participating in the trial, they were not the main person who shops for food in their household, or they had ever experienced a defined serious medical condition (heart failure, metastatic cancer, advanced kidney disease, advanced liver disease) or been given notice of a life expectancy of less than one year. We also excluded participants who had an upper arm circumference greater than 48 cm as cuffs larger than this were unavailable for the blood pressure monitors used in this study.

### Run-in

All potential participants meeting initial eligibility criteria were invited to commence a 5-week run-in phase during which baseline data were collected and further eligibility assessments were made. Participants first provided additional demographic and health data and were then directed to download and install the SaltSwitch OGS browser extension (details below). Participants were instructed to continue their usual grocery purchasing habits for the next 5 weeks online and while using the browser extension; 5 weeks was considered sufficient to assess commitment to online grocery shopping and capture baseline data. During run-in the browser extension simply captured data on grocery purchasing without making any recommendations about switches. Participants were also mailed a laboratory pathology form and asked to attend a local pathology provider and give a urine sample for measurement of sodium, creatinine, and potassium levels. They were also sent a link to complete a 24-hour dietary recall questionnaire [39].

Potential participants remained eligible if at the completion of the 5-week run-in they had: (1) completed at least two online shopping events; (2) purchased the equivalent of at least 500 g of food per person per day for their household; and (3) purchased products from at least five different food categories that contribute importantly to sodium intake in Australia [40]. If participants passed run-in they were sent a validated blood pressure monitor (iHealth^®^ FEEL BP5 or NEO BP5S Wireless Blood Pressure Monitor [41]), which directly transferred blood pressure readings to study investigators via a phone app, as well as detailed standardised instructions on use, including an on-demand video walkthrough. The receipt of blood pressure readings was taken as successful completion of run-in procedures. Neither completion of the dietary recall survey nor provision of the urine sample were required for a participant to advance to the main trial period.

### Randomisation and masking

Participants were randomised using a computer-generated sequence to either the control or the intervention group in a 1:1 ratio, stratified by age (≤ 50 years, > 50 years), sex (male, female) and baseline anti-hypertensive medication use (yes, no). The random allocation sequence was generated by a statistician, independent of both the support staff who recruited and managed participants and the study investigators. Randomisation triggered the SaltSwitch OGS browser extension to switch to either intervention or control mode. The nature of the intervention precluded masking, but all participant-facing material only mentioned that ‘this research study will investigate grocery shopping behaviour and how purchasing choices may change depending on the information being presented’. The mechanism of the intervention was only disclosed to those assigned to intervention after randomisation.

### Intervention and control

The SaltSwitch OGS intervention was a bespoke web browser extension that modified the user interface of a retailer’s online store, incorporating interpretive labelling and product swap functions. For each selected product a search for healthier alternatives in the same product category was made by referring to the Australian FoodSwitch database [42] (a large and regularly updated database of packaged products, see below). For the purposes of this study, “healthier” was defined as a product with lower sodium content and an equal or better nutrient profile according to the Australian Health Star Rating system (the government endorsed labelling system that summarises the overall healthiness of products based on their ingredient and nutrient composition) [43]. Healthier products, if in-stock, were displayed in order of sodium content as on-screen pop-ups. Information on sodium content was interpreted (via a simple colour-coded message) to assist with decision-making, with sodium thresholds set according to the United Kingdom’s traffic light front-of-pack labelling system [44]. Only products in stock at the relevant outlet were suggested as alternatives (Additional File 1, Supplementary Fig. 2). If the entire food category was high in sodium (e.g. salty snacks or processed meats), participants then were advised that the purchase of any item within that category should be reconsidered (Additional File 1, Supplementary Fig. 3). The web browser extension automatically recorded product selections, alternatives displayed, any switches made, and the full final list of groceries purchased. Participants assigned to the intervention group also received fortnightly advice by email about healthy behaviours that could assist in controlling blood pressure. Topics covered included the effect of sodium on blood pressure and cardiovascular disease risk, interpreting food labels, reducing sodium intake in different settings, and other relevant behavioural modifications.

For participants assigned to control, the SaltSwitch OGS browser extension simply recorded grocery purchases with the usual online grocery shopping process unchanged. Participants were not provided with any additional educational material.

### Food product data

Data describing the sodium content and nutrient profile of foods was obtained from the Australian FoodSwitch database which covers about 95% of the packaged food products typically purchased by Australian households [45]. The database contains data on more than 100,000 individual packaged products [42] and was continuously updated during the study using a web-scraping approach as described previously [38]. The web-scraping was performed quarterly to ensure that changes to product ranges were captured. As product ranges differ across the country, the data collection was done across a range of metropolitan and regional postcodes.

### Follow-up

Post-randomisation follow-up was for 12 weeks, during which all participants were asked to continue their usual frequency and volume of online shopping. All purchasing behaviour was recorded by the SaltSwitch extension. Both groups were prompted to measure blood pressure at weeks 6 and 12, though the study allowed participants to provide measures as often as they liked. Participants were asked to complete medication surveys and report serious adverse events at weeks 6 and 12, and to provide a further urine sample and complete a second dietary recall questionnaire at week 12. All participants received up to AUD$300 worth of grocery vouchers - $100 for downloading and activating the web-browser extension, and a further 2 x AUD$100 vouchers for providing baseline and week 12 blood pressure measurements, respectively.

### Outcomes

The primary outcome was the difference in mean sodium density (mg sodium per 1000 kcal of energy) of packaged groceries purchased over 12 weeks. The primary outcome data were captured automatically by the web browser extension. During occasions when the OGS data capture functionality was compromised (which was immediately detected by an in-built alert system) the participants were informed and asked to send their shopping receipts to the research team, while the data capture functionality was being repaired. Overall, 2419/2617 (92%) of receipts were collected by SaltSwitch OGS’s in-built functionality. Nutrition information (sodium and energy content) of products were matched to purchases using the FoodSwitch database, with data available for 8135/8158 (99.8%) unique packaged food products purchased by trial participants, and those (*n* = 23) not in the database identified through internet search and manual matching.

Secondary outcomes were difference in the change in (1) mean systolic and diastolic blood pressure measured by participants at home (mmHg); (2) mean spot urine sodium concentration; (3) mean spot urinary sodium: potassium ratio; and (4) diet quality quantified using the mean Dietary Approaches to Stop Hypertension (DASH) score, using a validated automated 24-hour diet recall administered online [39], as a summary of a number of dietary indicators. Blood pressure monitoring was supported through the provision of instructions on optimal methods for home blood pressure measurement. Any fees associated with pathology tests were paid by trial investigators via prior billing arrangements established with major pathology providers.

Exploratory outcomes included: (1) hypertension control defined as both systolic blood pressure < 130 mmHg and diastolic blood pressure < 80 mmHg; (2) anti-hypertensive medication use; and (3) mean salt intake in grams per day calculated from spot urine collections using estimating equations based on the INTERSALT study [46]. We also assessed impacts on purchasing habits and product choice, including number and value of shopping receipts and number of product switches recorded.

Safety was assessed on the basis of serious adverse events, including, but not limited to, life-threatening events, intercurrent hospitalisation, persistent or significant disability or incapacity, congenital anomaly or birth defect, and presentation for acute medical care. A full data and safety monitoring committee was not convened as the intervention was considered to be at low risk of causing or contributing to any adverse outcomes; rather a practicing cardiologist was appointed as an independent safety monitor [38].

### Sample size

A minimum sample size of 127 was calculated as required to detect a 20% or greater difference in the sodium density of purchased products in the intervention compared to the control group. This sample size was projected to provide 90% power with a two-sided alpha of 0.05. The power estimate assumed a mean baseline sodium density of online grocery shopping of 1080 mg/1000 kcal with a standard deviation of 350 mg/1000 kcal, derived from grocery purchasing data from participants enrolled in this trial between July 2021 and August 2022 (i.e. before the primary outcome of interest was revised), as well as unpublished analysis of data from *n* = 7,186 households in the NielsenIQ Australian Homescan panel over 12 months in 2018. We also allowed for a dropout from the intervention of up to 10% in calculating the minimum sample size. The effect size was considered likely conservative based on the findings of a New Zealand study using a similar intervention delivered as a smartphone app developed for use in physical stores [47].

### Analysis

All analyses were conducted as per the previously published statistical analysis plan (https://osf.io/347ps/). To assess the primary outcome, food purchasing data were grouped into periods defined by run-in, weeks 1–4, weeks 5–8 and weeks 9–12 for each participant, with foods and beverages exempted from displaying nutritional information (e.g. single ingredient foods) excluded. We used linear mixed models with all follow-up sodium density data as the dependent variable, follow-up time (week 1–4, week 5–8 and week 9–12), randomised allocation (intervention and control), baseline (run-in) sodium density and the interaction between follow-up time and intervention group included in the model. Participants were included as a random intercept to account for the multiple follow-up data points for each participant. The p-value for the primary outcome is based on the mixed model assessing difference in mean sodium density of purchased products between groups, adjusted for baseline sodium density but without an interaction term between follow-up time and intervention group assignment. Subgroup analyses by sex, age, and baseline use of anti-hypertensive medication were conducted to investigate if there were any different effects among these subgroups. Sensitivity analyses including additional covariates (ethnicity, income and education) were conducted to adjust for the potential impact of these covariates. Possible non-linear effects of the intervention were also explored by comparing effect sizes across each 4-week follow-up period. Household size was not adjusted for in these analyses as sodium density, as a characteristic of the product itself, is not affected by the number of people in a household.

Effects on blood pressure were investigated using the same methods as were employed for the primary outcome, while analysis of covariance was used for all other secondary outcomes which were assessed using baseline and 12 weeks measures. There was no imputation with analyses done on complete-case records since primary outcome data was 99% complete, blood pressure data was 94% complete, and data for other secondary outcomes was also largely complete. All statistical analyses were performed using R version 4.2.3 and Rstudio version 2023.12.1 Build 402. Packages “gtsummary” and “lme4” were used to summarize data and conduct statistical analyses.

### Role of the funding source

This study was funded by the Australian Government Medical Research Future Fund. The funder had no role in study design, the collection, analysis or interpretation of data, the writing of the report, and the decision to submit a paper for publication.

## Results

**Fig. 1:**
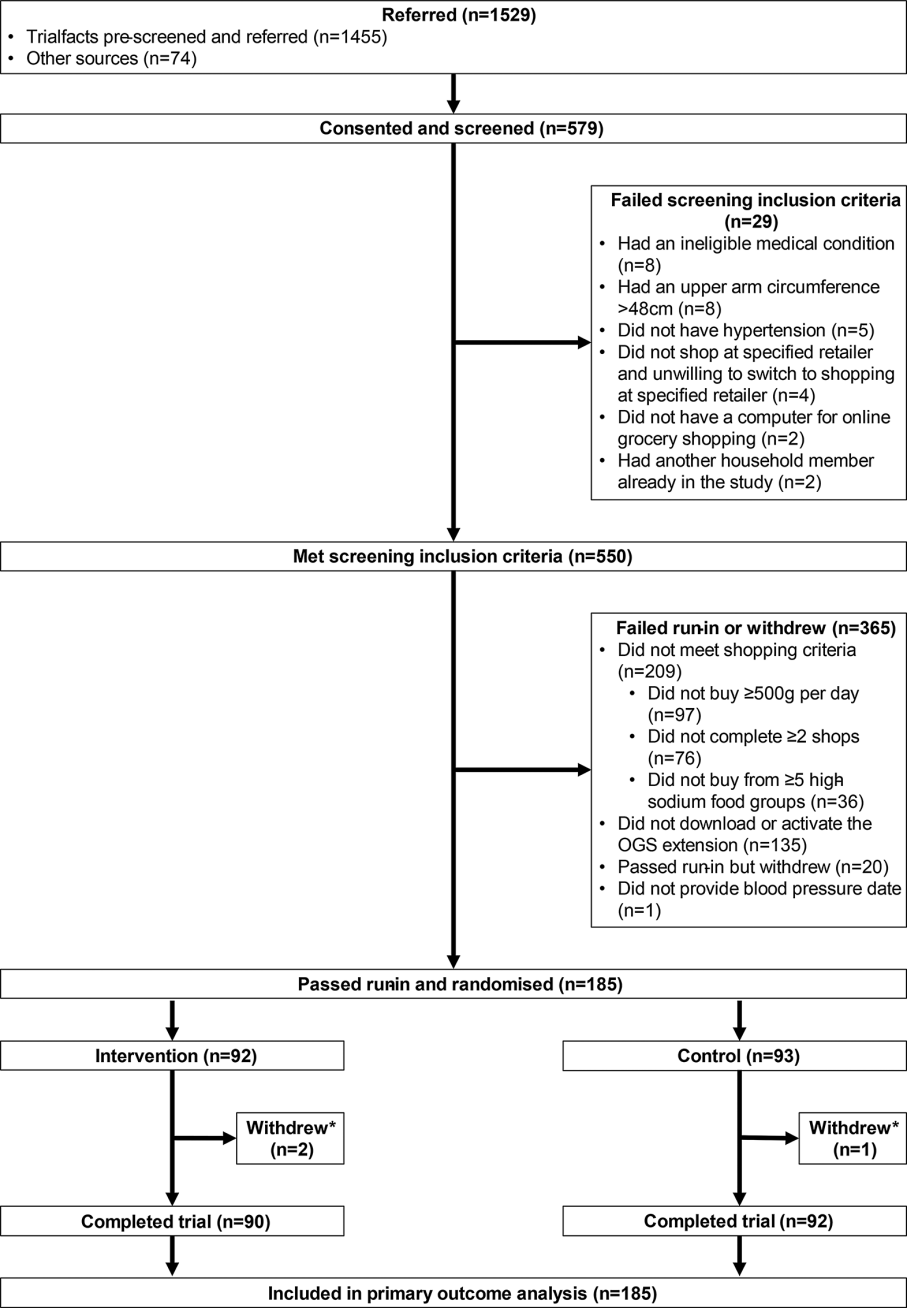
Trial flowchart. *The three participants who withdrew from the trial did so at weeks 3, 8, and 12 of follow-up, and gave consent for data already collected during the trial to be used

There were 1,529 potential participants referred from recruitment sources with the majority (*n* = 1,455; 95%) from the recruitment company Trialfacts (Fig. 1), from which 579 consented and 550 were assessed as eligible to commence run-in. Of these, 365 failed run-in, due mostly either to failure to meet purchase quantities of total groceries or high sodium food categories (*n* = 209), or failure to download the SaltSwitch OGS web browser extension (*n* = 135). From July 2021 to June 2023, there were 185 participants randomised with 3 participants not completing follow-up (2 intervention and 1 control).

### Baseline characteristics

Baseline demographic and disease history characteristics were similar across the randomised groups. Participants were on average 56 years of age, 64% were women, 91% were the main cook in the household, and 70% lived with other adults or children (Table 1). Most had BMI > 25 kg/m^2^ (91%), were White (89%) and had completed tertiary education (88%). Anti-hypertensive medication use was frequent (83%) but few reported a history of cardiovascular disease (4.9%) or diabetes (18%). Less than half (41%) had received advice to reduce their salt intake.

**Table 1:** Participants characteristics at study baseline

Characteristic	Overall, *n* = 185	Intervention group, *n* = 92	Control group, *n* = 93
Age, years (mean ± SD)	56.0 (11.0)	55.5 (11.2)	56.5 (10.8)
Female (n, %)	119 (64%)	59 (64%)	60 (65%)
BMI, kg/m^2^ (mean ± SD)	34.2 (7.8)	34.7 (8.0)	33.8 (7.6)
Race (n, %)			
Aboriginal and/or Torres Strait Islander	4 (2.2%)	3 (3.3%)	1 (1.1%)
Asian	11 (5.9%)	8 (8.7%)	3 (3.2%)
White	164 (89%)	77 (84%)	87 (94%)
Other	6 (3.2%)	4 (4.3%)	2 (2.2%)
Highest education completed (n, %)			
Primary/secondary school	22 (12%)	12 (13%)	10 (11%)
TAFE or Trade Certificate or Diploma	59 (32%)	29 (32%)	30 (32%)
University and above	103 (56%)	51 (55%)	52 (56%)
Other	1 (0.5%)	0 (0%)	1 (1.1%)
Employment (n, %)			
Employed	88 (48%)	41 (45%)	47 (51%)
Unemployed	92 (50%)	49 (53%)	43 (46%)
Other	5 (2.7%)	2 (2.2%)	3 (3.2%)
Income (n, %)			
<=41,599	39 (21%)	16 (17%)	23 (25%)
> 41,599 to < = 103,999	75 (41%)	37 (40%)	38 (41%)
> 103,999	53 (29%)	29 (32%)	24 (26%)
Prefer not to answer	18 (9.7%)	10 (11%)	8 (8.6%)
Number of adults in the household (mean ± SD)	1.96 (0.85)	1.96 (0.77)	1.96 (0.93)
Number of children in the household (mean ± SD)	0.36 (0.75)	0.38 (0.72)	0.34 (0.77)
Participant is the main cook (n, %)	168 (91%)	81 (88%)	87 (94%)
Smoking (n, %)			
Yes	7 (3.8%)	3 (3.3%)	4 (4.3%)
Previously	16 (8.6%)	10 (11%)	6 (6.5%)
No	162 (88%)	79 (86%)	83 (89%)
Have received advice to reduce salt intake (n, %)	76 (41%)	41 (45%)	35 (38%)
Previous cardiovascular disease (n, %)	9 (4.9%)	2 (2.2%)	7 (7.5%)
Have type 2 diabetes (n, %)	34 (18%)	16 (17%)	18 (19%)
Taking anti-hypertensive medication	153 (83%)	76 (83%)	78 (84%)

### Effect of the intervention on sodium density of purchased products

**Fig. 2:**
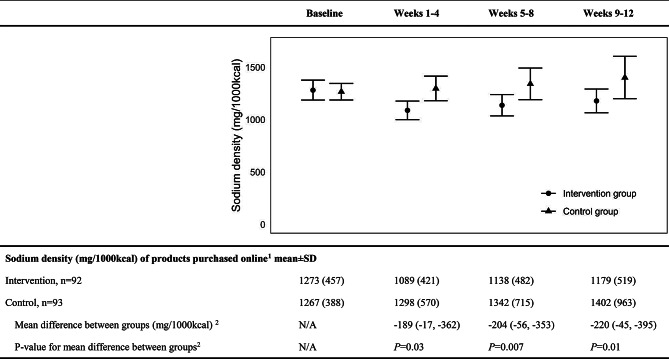
Effect of SaltSwitch OGS intervention on sodium density of foods and beverages purchased online. ^1^Based on shopping receipts captured during the study period. Sodium density of purchased products was calculated based on receipts that were grouped into 4-week periods, i.e. baseline (study run-in) and follow-up weeks 1–4, 5–8, and 9–12 and sodium density of purchased products calculated within each period. In the figure, the error bars are 95% confidence intervals of sodium density of purchased products of each group at each time point. ^2^Analyzed using linear mixed models assessing difference in mean sodium density of purchased products between groups, adjusted for baseline sodium density and with an interaction term between week and group. Mean differences and p-values shown at each time point were estimated using the margins package in R version 4.2.3 and Rstudio 2024.04.2 Build 764

Mean baseline sodium density of product purchases was 1273 mg/1000 kcal for the intervention group and 1267 mg/1000 kcal for the control group during run-in (Fig. 2). Over the 12-week post-randomisation period, the mean sodium density of product purchases was − 204 (95%CI, -352 to -156) mg/1000 kcal lower in the intervention group compared to the control group (*P* = 0.01). Adjustment for race, education, and income did not appreciably alter the observed effect (-202, 95%CI -359 to -45 mg/1000 kcal). The reduction in sodium density was apparent during weeks 1–4 of follow-up (-189, 95%CI -362 to -17 mg/1000 kcal) and was similar at weeks 5–8 (-204, 95%CI -353 to -56 mg/1000 kcal) and weeks 9–12 (-220, 95%CI -395 to -45 mg/1000 kcal) (p interaction = 0.74). Intervention effects were also similar in subgroups defined by sex, age, and baseline use of anti-hypertensive medication (p heterogeneity all > 0.1) (Additional File 1, Supplementary Table 2).

### Effect of the intervention on shopping habits and product choice

During the 5-week run-in, intervention and control groups both completed about six shopping events with a mean value of around AUD$120 each. Following randomisation, there were two intervention group participants that did not purchase any food online during follow-up. Otherwise, there were similar rates of shopping and similar average spend to that observed during run-in (Table 2**).** Of the 90 participants in the intervention group who completed at least one shopping event post-randomisation, 86% made at least one switch to a lower sodium product as suggested by the SaltSwitch intervention (Table 3). Of the 960 shopping events recorded, at least one such switch was made in one-third (35%), with an average 1.9 switches in each shopping event where any switch was made. Switching activity was constant throughout the 12-week intervention period (Additional File 1, Supplementary Fig. 4).

**Table 2:** Online groceries purchased by trial participants based on shopping receipts [1]

	Intervention (*n* = 92)	Control (*n* = 93)
**Baseline (run-in period) of 4 weeks**		
Shopping receipts recorded per participant		
Mean ± SD	6.3 (2.7)	6.7 (3.7)
Median (IQR)	6.0 (4.0, 8.0)	6.0 (4.0, 9.0)
Value ($) of shopping receipts		
Mean ± SD	117.8 (64.9)	124.0 (68.7)
Median (IQR)	106.2 (66.8, 157.0)	104.5 (76.1, 164.5)
**Follow-up over 12 weeks**		
Did not complete any online grocery shopping during follow-up, n(%)	2 (2%)	0 (0%)
Shopping receipts recorded per participant		
Mean ± SD	10.7 (4.0)	11.2 (4.6)
Median (IQR)	10.0 (8.0, 13.0)	12.0 (7.0, 15.0)
Value ($) of shopping receipts		
Mean ± SD	113.8 (63.9)	128.5 (78.6)
Median (IQR)	97.2 (67.1, 149.4)	108.5 (74.4, 165.7)

^1^Non-food products were excluded from analyses. Results were similar when non-food products were included with similar purchasing patterns between groups but higher total expenditure per shopping event (not shown)

**Table 3:** Changes in grocery selection for participants in the OGS trial intervention group during study follow-up [1, 2]

Shopping characteristics influenced by OGS SaltSwitch	
**Number who did any online grocery browsing during the follow-up period**,** n**	90
Number and proportion of participants who made at least one switch to a lower sodium product during study follow-up.	77 (86%)
**Total browsing events recorded**,** n**	960
Number of browsing events where there was ≥ 1 switch in product selection from the initial higher sodium product to a lower sodium product recommended by SaltSwitch, n (%)	332 (35%)
**Average number of items browsed within each browsing event where participants made ≥ 1 switch**	10 ± 8
Average number of switches from initial high sodium product to a lower sodium product recommended by SaltSwitch within browsing events where switches were made	1.9 (1.5)

^1^Each time a participant logged into their online grocery shop and clicked on products, the OGS automatically recorded the activity as a browsing event. Each product the participant clicked on was recorded as a ‘browsed item’

^2^If during a browsing event the participant switched their product selection from those higher sodium to those lower in sodium as recommended by the SaltSwitch intervention, these were also recorded by the OGS application as part of its data tracking process

### Effect of the intervention on other secondary, exploratory and safety outcomes

There were no detectable effects of the intervention on blood pressure, urine sodium concentration, urine sodium: potassium ratio, and diet quality (Additional File 1, Supplementary Tables 3 and 3). Similarly the intervention did not affect hypertension control, use of anti-hypertensive medication, or estimated salt intake (not shown). There were four serious adverse events recorded (one in intervention and three in the control), but none were related to the study intervention.

## Discussion

This study showed that a simple modification to the user interface of an online supermarket can reduce the amount of sodium purchased from packaged foods. The size of the reduction, about 200 mg of sodium per day (based on unpublished data from this study and NielsenIQ consumer panels, in which households purchased approximately 1000 kcal of energy from pre-packaged food per day) equates to about 500 mg of salt, which represents about 5% of current average intake for Australian adults [48]. While relatively small in magnitude, this reduction is estimated to translate into a reduction in systolic blood pressure of about 1mmHg among patients with hypertension, which would lower the risk of stroke, heart attack, kidney disease and heart failure [49].

The positive finding for the SaltSwitch OGS intervention is important because there is no other salt reduction program in Australia that has proved effective to date. The flagship national salt reduction effort is the ‘Healthy Food Partnership’ led by the federal government, which has set salt reformulation benchmarks for 32 packaged food categories and is now reaching the conclusion of a 4-year implementation period. Prior modelling studies have indicated that full implementation of the Healthy Food Partnership targets would deliver a reduction in salt intake of about 268 mg (~ 100 mg sodium) per person [50]. This is half of what was observed for the SaltSwitch OGS intervention, though in practice no Healthy Food Partnership reformulation targets have been met to date, and the current projection from government is for the Healthy Food Partnership to have minimal effect on salt consumption levels [51].

For patients with hypertension managed by the health care system clinical guidelines recommend salt reduction. Among our hypertensive trial participants, however, less than half reported having received advice to reduce salt intake, while clinical counselling to reduce salt intakes is known to be of limited effectiveness [52]. Switching use of regular salt for potassium-enriched salt in the home has been showed effective for disease prevention in settings where a large proportion of dietary sodium derives from discretionary salt [53], but potassium-enriched salt is not widely recommended or used in Australia.

Against this background, there is an urgent need for innovative ways of reducing sodium intake. The SaltSwitch OGS browser extension combined a series of elements that have proved individually effective elsewhere in improving the healthiness of consumer food purchases. These include the use of colour-coded and interpretive labelling [17–19, 24], the use of warnings [54], providing an easy to use swap function [35, 36], and delivering the intervention at the point-of-sale [24]. The collective use of these elements within the SaltSwitch OGS intervention maximised the likelihood of success though the joint use of multiple strategies precludes us from defining the separate contribution made by each. It is likely that all were important, and some may have acted synergistically though the importance of each element may have varied between individual users. Effects would likely also be augmented if promotions, particularly price-based, were used as complementary levers to encourage healthier switches [55]. On the other hand, product prices, which were not considered in the display of alternative options in the current iteration of this intervention, are likely to have been influential in decision-making and regardless of product healthiness [56]. The impact of the intervention, prices and other factors on the selection of products will be assessed through a qualitative exploration of participant experiences during the SaltSwitch OGS trial.

Sustained impact over 12 weeks was anticipated since online grocery shopping sites typically store prior shopping lists, meaning that once a switch to a lower sodium product was made it would be readily accessible in subsequent shops. However, we observed that broadly similar numbers of individual product switches were made across the first, middle and final four-week periods of the 12-week intervention. This indicates that some participants were consistently directly navigating to and selecting specific products throughout the 12 weeks before making a switch, rather than relying on saved lists. Consistency in the number of switches made by participants may be due to them returning to previous purchases initially with repeated successfully encouragement to switch to healthier alternatives, many of the product types being selected for switches not being frequently purchased across the period, and/or new switches being tried while previous ones were not continued. Though this may suggest that habits were hard to shift or that changes made were not sustained, it is encouraging that participants continued to engage with the intervention over the 12-week study period. It is currently unclear whether the positive effects seen would remain consistent, compound or decrease with a longer duration of use, however. Greater early effects were observed in the prior study most comparable to ours, the ‘Dietary Intervention in e-Shopping Trial,’ [36] which showed a significant reduction in mean saturated fat purchases in an online grocery setting (median follow-up 35 days).

Several other studies involving real-world interventions in online supermarkets have reported success in enabling healthier product choices by consumers, including those that utilised automated product swap functions or interpretive food labels [35–37]. In addition to enabling switching, it is also likely that online tools of this type can defer some purchases entirely. For example, in our trial when switching was not possible because the entire category was high sodium, some participants may have heeded the warning message provided and chosen not to purchase from the category at all. This form of action was not able to be recorded by the data tracking function in our web browser extension and the extent to which switches versus deferred purchases drove the observed reduction in sodium is unknown.

Our intervention did not improve overall diet quality, which is unsurprising given it focused on sodium alone. Sodium content is only one part of the measures of diet quality used, which also incorporates 8 other nutritional targets [57]. While the success of the SaltSwitch OGS intervention suggests that a strategy that integrates and targets switches for specific populations may be effective, further testing of the intervention based upon a wider set of diet quality indicators and amongst the general population will be required to assess its potential to deliver broader improvements in dietary patterns.

The greatly reduced sample size was a major weakness of our study since it meant we only had statistical power to detect effects on sodium purchases and could not robustly test effects on clinical outcomes such as blood pressure and urine electrolyte concentrations. It is likely that reduced sodium purchases will translate into clinical benefits, but direct evidence of clinical effects would have been much more compelling in regard to driving uptake of SaltSwitch OGS by food retailers. This is important because in practice an intervention like SaltSwitch OGS is only likely to be successful if incorporated directly into the technology platforms of major retailers. While our strategy of delivering the functionality independently as a browser extension was able to be implemented, it required ongoing maintenance to integrate with the retailer’s online grocery site, which was continually updated. The need to download and install a browser extension was also a reason why many potential trial participants did not join the study and would likely be a major impediment to scaling use among the wider community. These issues could be overcome if the intervention functionality was embedded into a retailer’s website.

The generalisability of the trial result is also uncertain since only motivated individuals were likely to pass study run-in. However, estimated 24-hour salt intake, using the INTERSALT with potassium equation [58], of 8.7 g per person per day across both the intervention and control groups during run-in (results not shown) is comparable to that found amongst Australians generally and those with hypertension (8.7 g and 8.8 g, respectively) [48], suggesting that participants may be representative of the wider population. The comparable effects on sodium purchases observed in subgroups also provide some reassurance about likely similar effects across different population groups though statistical power to detect difference between subgroups was limited. The study assessed only people with self-reported diagnosis of elevated/high blood pressure and/or use of anti-hypertensive medication, with 6.8 million Australian adults (one-third) having hypertension [59]. A prior study of SaltSwitch functionality tested as a smartphone application for use in-store done in New Zealand also showed reduced sodium purchases among individuals with cardiovascular disease [47].

Key strengths of the study were the randomised controlled design and the high completion rate, which minimised the risk of bias due to dropouts. Likewise, basing the primary outcome on recorded shopping receipts provided for a highly objective measure of effect. The data collected on the average value of shopping receipts suggests that the intervention is likely to be revenue neutral, which is likely to be an important consideration for retailers considering the adoption of interventions such as this [60], although other factors such as supplier relationships and contracts may also be a barrier to uptake by retailers.

## Conclusions

In conclusion, our study is an important addition to the growing body of evidence demonstrating the potential for innovative health interventions designed to leverage off the growth in online grocery shopping. Automated switching interventions that utilise methods such as those deployed by SaltSwitch OGS appear to have significant potential to promote healthier product purchasing at low cost for large numbers. The platform we have developed could easily be adapted to reduce the purchasing and consumption of other nutrients of concern like added sugar, or incorporate multiple components to promote a generally healthier diet. There is also clear potential to offer customers switching solutions tailored to their specific health needs. Future research now needs to tackle the major challenge of how to translate these promising findings into practice.

## Electronic supplementary material

Below is the link to the electronic supplementary material.

Supplementary Material 1: Additional File 1. Description of data: Provides explanatory material and additional results, as referenced in text.

## Data Availability

Deidentified participant data that underlie any results reported by study investigators, as well as study documents such as data dictionaries, information and consent forms, case report forms and statistical code, may be shared after contacting study investigators, subject to relevant policies of the host organisation.

## Abbreviations

DASH: Dietary Approaches to Stop Hypertension
SaltSwitch OGS: SaltSwitch for Online Grocery Shopping
WHO: World Health Organization
