# Supplementary material for: Effectiveness of an online food shopping intervention to reduce salt purchases among individuals with hypertension – findings of the SaltSwitch Online Grocery Shopping (OGS) randomised trial
Source: Int J Behav Nutr Phys Act. 2024 Dec 30;21:148. doi: 10.1186/s12966-024-01700-9 (PMC11687014; doi:10.1186/s12966-024-01700-9)
Supplement: Supplementary file 1 — Supplementary Material 1: Additional File 1. Description of data: Provides explanatory material and additional results, as referenced in text. [file 12966_2024_1700_MOESM1_ESM.docx]

**Supplementary Figure 1. Trial Schema**

**
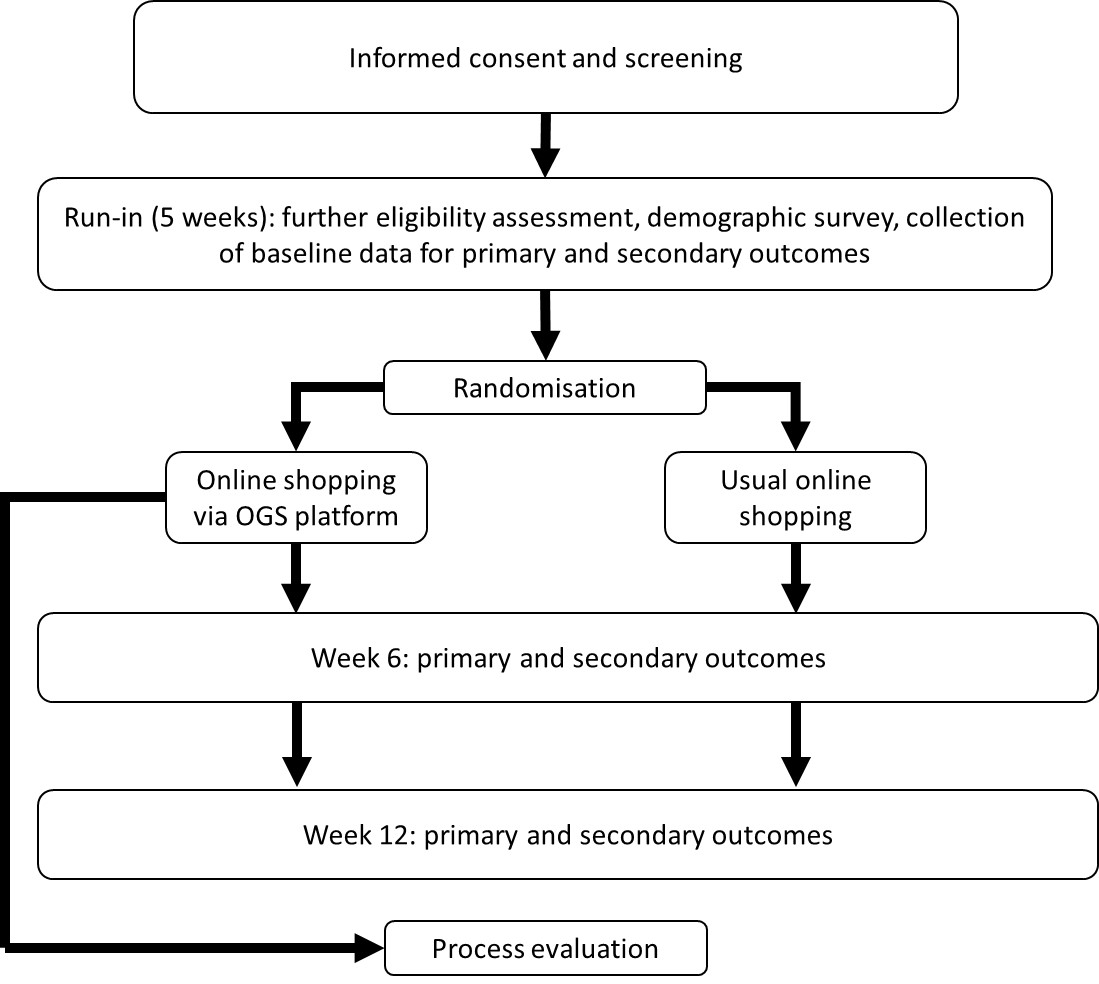
**

**Supplementary Figure 2 Example of OGS extension pop-up**


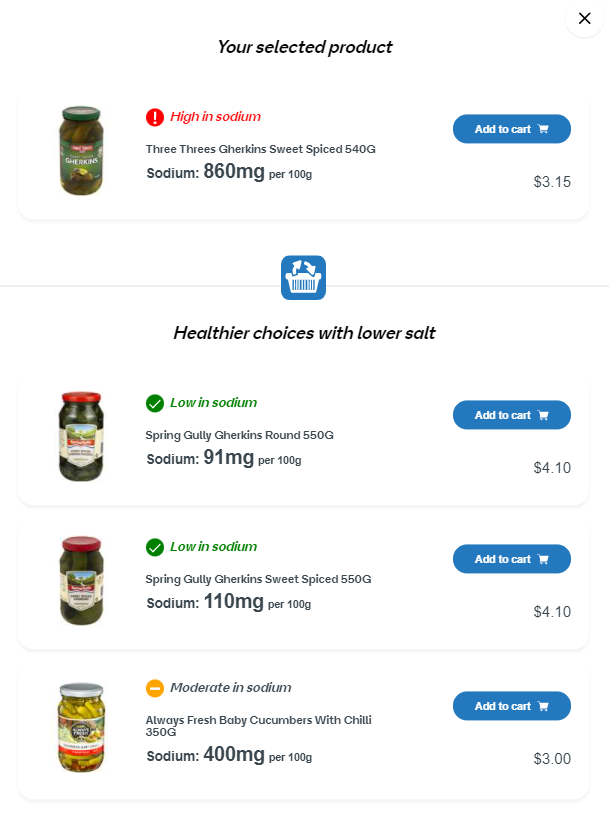


**Supplementary Figure 3 OGS extension (intervention mode) flow chart.**


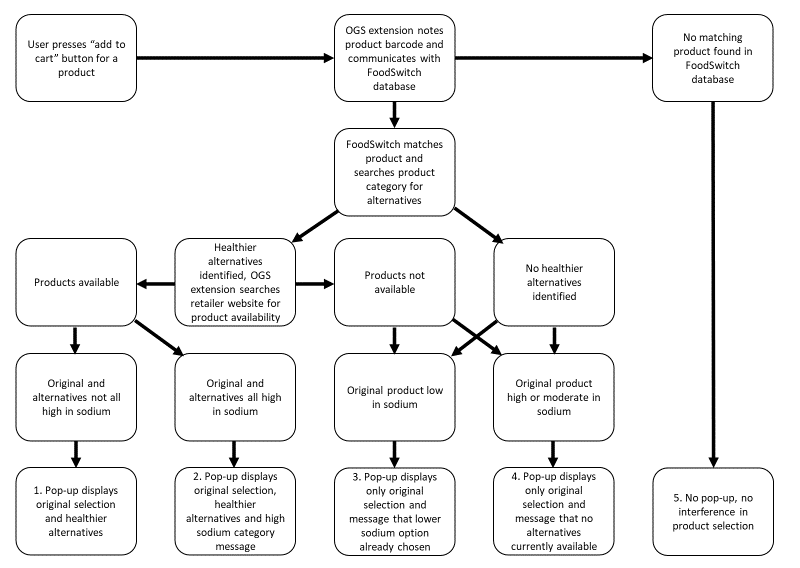


**Supplementary Table 1. Study activities**

| **Activity** | **Run-in period/baseline** | **Main trial period (week 6)** | **Main trial period (week 12)** | **Process evaluation (post-week 12)** |
| --- | --- | --- | --- | --- |
| **Demographic survey** | X |  |  |  |
| **Physical activity survey** | X | X | X |  |
| **Medication use survey** | X | X | X |  |
| **Online groceries purchases*** | X | X | X |  |
| **Blood pressure measurements*** | X | X | X |  |
| **Dietary recall questionnaire** | X |  | X |  |
| **Urine sample** | X |  | X |  |
| **Telephone interview** |  |  |  | X |

* Data are continuously collected, with prompts sent during run-in and at weeks 6 and 12.

**Supplementary Table 2. Differences in treatment effects** **of SaltSwitch OGS intervention on sodium density of foods and beverages in key subgroups**

|  | **Sodium density (mg/1000kcal) of purchased groceries over 12 weeks of the study^1^** | | | ***P* for heterogeneity^2^** |
| --- | --- | --- | --- | --- |
| **Participant Characteristics** | **Control Group** | **Intervention Group** | **Differences in means between intervention and control groups** |  |
| Age ≤50years | 1579 (1360, 1799) | 1156 (945, 1366) | -424 (-727, -120) | 0.11 |
| Age >50 years | 1269 (1151, 1388) | 1129 (1007, 1251) | -140 (-310, 30) |  |
|  |  |  |  |  |
| Women | 1362 (1233, 1492) | 1223 (1089, 1356) | -139 (-325, 47) | 0.27 |
| Men | 1300 (1126, 1475) | 987 (812, 1162) | -313 (-560, -66) |  |
|  |  |  |  |  |
| Not using anti-hypertensive medication | 1170 (876, 1465) | 1070 (761, 1379) | -100 (-527, 327) | 0.61 |
| Using anti-hypertensive medication | 1365 (1252, 1477) | 1145 (1031, 1259) | -220 (-379, -60) |  |

^1^Sodium density of purchased products was calculated based on receipts captured during the study period.

^2^Heterogeneity in treatment effects across subgroups were tested by fitting an interaction term between the characteristics of interest and treatment assignment.

**Supplementary Figure 4.** The number of switches made by trial participants from an initial higher sodium to a lower sodium product recommended by SaltSwitch separately for weeks 1-4, 5-8, and 9-12 of follow-up. The mean (SD) switches per browsing event were 0.8 (1.5), 0.7 (1.2) and 0.5 (1.0) in weeks 1-4, 5-8, and 9-12 weeks of follow-up, respectively.


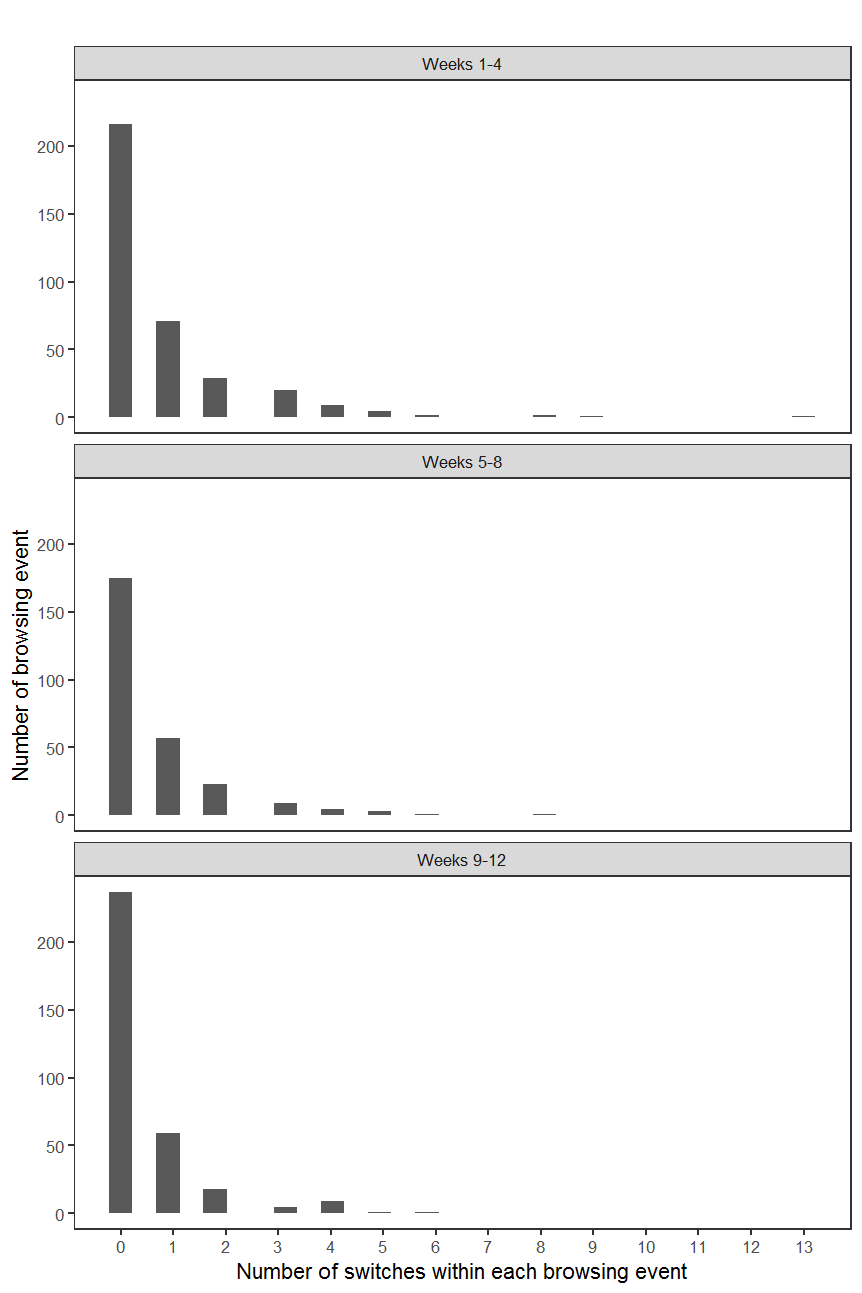


**Supplementary Table 3. Effects of SaltSwitch OGS on systolic and diastolic blood pressure^1^**

|  | **Intervention** | | | |
| --- | --- | --- | --- | --- |
| **Groups** | **Run-in** | **Weeks 1-4** | **Weeks 5-8** | **Weeks 9-12** |
|  | *SBP, mmHg^2^* | | | |
| Intervention, n=82 | 135 (13) | 132 (13) | 133 (14) | 134 (13) |
| Control, n=91 | 137 (14) | 134 (13) | 136 (14) | 135 (14) |
| Mean difference between groups, mmHg | N/A | -0.8 (-3.3, 1.8) | -0.4 (-2.8, 1.9) | -0.1 (-2.5, 2.2) |
| P-value for mean difference between groups, mmHg | N/A | *P=*0.56 | *P=*0.71 | *P=*0.92 |
|  | *DBP, mmHg^2^* | | | |
| Intervention, n=82 | 83 (8) | 81 (10) | 82 (8) | 83 (9) |
| Control, n=91 | 83 (8) | 83 (8) | 83 (8) | 83 (7) |
| Mean difference between groups, mmHg | N/A | -1.2 (-2.7, 0.4) | -0.5 (-1.9, 0.9) | 0.1 (-1.3, 1.6) |
| P-value for mean difference between groups, mmHg | N/A | *P=*0.15 | *P=*0.47 | *P=*0.88 |

^1^Participants were provided instructions on optimal method for home blood pressure measurement using the same model of blood pressure monitor provided by the research team. They were asked to use the monitors as regularly as they wanted to, and the mean (SD) number of readings provided by participants were 124 (99) and 156 (113) in the intervention and control groups, respectively.

^2^Readings were grouped into 4-week periods, i.e. baseline (study run-in) and follow-up weeks 1-4, 5-8, and 9-12 and blood pressure result for a participant in this period calculated as a mean, before the mean (SD) for each group within each period was calculated.

^3^Analyzed using linear mixed models assessing difference in mean blood pressure between groups, adjusted for baseline blood pressure and with an interaction term between week and group. Mean differences and p-values shown at each time point are the estimated mean differences and p-values using the margins package in R version 4.2.3 and Rstudio 2024.04.2 Build 764.

## **Supplementary Table 4. Effects of SaltSwitch OGS on urine sodium, sodium to potassium ratio, and self-reported diet quality**

|  | **Intervention group** | | **Control group** | |  | |
| --- | --- | --- | --- | --- | --- | --- |
| **Characteristic** | **Run-in** | **Week-12** | **Run-in** | **Week-12** | **Mean differences between groups (95% CI)^3^** | **p-value^3^** |
| Spot urine sodium concentration, mmol/L^1^ | 79 (42) | 88 (51) | 88 (44) | 88 (50) | 3 (-12; 19) | 0.67 |
| Spot urine sodium:potassium concentration ratio^1^ | 1.7 (1.0) | 1.8 (1.5) | 1.8 (1.1) | 1.6 (0.9) | 0.2 (-0.2; 0.7) | 0.23 |
| DASH diet score^2^ | 3.9 (0) | 3.9 (0) | 3.8 (0) | 3.9 (0) | -0.1 (-0.2; 0.0) | 0.19 |

^1^Particiapnts were asked to provide spot urine samples to clinical pathology labs to assess urine concentrations of sodium and potassium. Results shown are based on n=67 participants, i.e. ~70% of participants in both groups who provided spot urine samples during run-in and during week 12 of the intervention.

^2^Participants were asked to complete an automated and previously validated online 24-hour diet recall, which was used to calculate the Dietary Approaches to Stop Hypertension Score as a measure of overall diet quality according to published methods (1). The continuous DASH score has a minimum and maximum possible value of 0 and 9, with higher values indicating healthier diet quality. Results shown are based on n=74 participants and n= 81 participants in the intervention and control groups who completed the 24-hour dietary recall during run-in and week-12 of the intervention, i.e. 80-90% of participants completed the dietary recall.

^3^Estimated Mean differences and p-values between groups were assessed using analysis of covariance, adjusting for baseline values of each variable.

**References**

1. Mellen PB, Gao SK, Vitolins MZ, Jr DCG. Deteriorating Dietary Habits Among Adults With Hypertension: DASH Dietary Accordance, NHANES 1988-1994 and 1999-2004. Arch Intern Med. 2008;168(3):308-14.
